# Renewed calls for abortion-related research in the post-Roe era

**DOI:** 10.3389/fpubh.2023.1322299

**Published:** 2023-12-18

**Authors:** Sophie L. Schott, April Adams, Ryan J. Dougherty, Taylor Montgomery, Folasade C. Lapite, Faith E. Fletcher

**Affiliations:** ^1^Center for Medical Ethics and Health Policy, Baylor College of Medicine, Houston, TX, United States; ^2^Department of Obstetrics and Gynecology, Baylor College of Medicine, Houston, TX, United States; ^3^Center for Medical Ethics and Health Policy, Baylor College of Medicine, Houston, TX, United States; ^4^Harvard Pilgrim Health Care Institute, Harvard Medical School, Boston, MA, United States; ^5^Department of Population Medicine, Harvard Medical School, Boston, MA, United States; ^6^School of Medicine, Tulane University, New Orleans, LA, United States

**Keywords:** abortion, research ethics, health equity, reproductive health research, population vulnerability

## Abstract

Nearly 50 years after Roe versus Wade, the United States Supreme Court’s decision in Dobbs versus Jackson Women’s Health Organization unraveled the constitutional right to abortion, allowing individual states to severely restrict or ban the procedure. In response, leading medical, public health, and community organizations have renewed calls for research to elucidate and address the burgeoning social and medical consequences of new abortion restrictions. Abortion research not only includes studies that establish the safety, quality, and efficacy of evidence-based abortion care protocols, but also encompasses studies on the availability of abortion care, the consequences of being denied an abortion, and the legal and social burdens surrounding abortion. The urgency of these calls for new evidence underscores the importance of ensuring that research in this area is conducted in an ethical and respectful manner, cognizant of the social, political, and structural conditions that shape reproductive health inequities and impact each stage of research—from protocol design to dissemination of findings. Research ethics relates to the moral principles undergirding the design and execution of research projects, and concerns itself with the technicalities of ethical questions related to the research process, such as informed consent, power relations, and confidentiality. Critical insights and reflections from reproductive justice, community engagement, and applied ethics frameworks have bolstered existing research ethics scholarship and discourse by underscoring the importance of meaningful engagement with community stakeholders—bringing attention to overlapping structures of oppression, including racism, sexism, and ways that these structures are perpetuated in the research process.

## Renewed calls for abortion-related research in the post-Roe era

Nearly 50 years after *Roe* versus *Wade,* the United States Supreme Court’s decision in *Dobbs* versus *Jackson Women’s Health Organization* unraveled the constitutional right to abortion, allowing individual states to severely restrict or ban the procedure. In response, leading medical, public health, and community organizations have renewed calls for research to elucidate and address the burgeoning social and medical consequences of new abortion restrictions (1–5). Abortion research not only includes studies that establish the safety, quality, and efficacy of evidence-based abortion care protocols, but also encompasses studies on the availability of abortion care, the consequences of being denied an abortion, and the legal and social burdens surrounding abortion (6, 7). The urgency of these calls for new evidence underscores the importance of ensuring that research in this area is conducted in an ethical and respectful manner, cognizant of the social, political, and structural conditions that shape reproductive health inequities and impact each stage of research—from protocol design to dissemination of findings.

Research ethics relates to the moral principles undergirding the design and execution of research projects, and concerns itself with the technicalities of ethical questions related to the research process, such as informed consent, power relations, and confidentiality (8). Critical insights and reflections from reproductive justice, community engagement, and applied ethics frameworks have bolstered existing research ethics scholarship and discourse by underscoring the importance of meaningful engagement with community stakeholders—bringing attention to overlapping structures of oppression, including racism, sexism, and ways that these structures are perpetuated in the research process (9–19).

Scholars have critiqued traditional research ethics models for being too narrowly focused on investigator expertise and conventional measures of scientific validity. While helpful in some scenarios, this narrow focus can obscure the needs of minoritized communities with structural vulnerabilities and silence their voices across the research continuum. In essence, research can only be ethical when it prioritizes equity, justice, and respect for groups burdened with the potential to be most harmed during the research process.

Considering the heightened challenges posed by the post-*Roe* era, the commentary that follows is a call for researchers, research institutions, funding agencies, Institutional Review Boards (IRBs) and other regulatory bodies to safeguard against potential research-related harms by (1) prioritizing the needs, concerns, and preferences of populations burdened by social and structural vulnerabilities (20) promoting reproductive justice-oriented, community-engaged scholarship, and (21) providing evidence-based training and robust support for researchers. Given the history of medical exploitation and reproductive violence in communities with structural vulnerabilities, ethical and respectful research in the post-*Roe* environment requires prioritizing the voices of the most marginalized to mitigate iatrogenic research harms and promote reproductive health equity (20).

## The social, ethical, and legal complexities of abortion-related research

Early research on abortion focused on instances in which pregnancy terminations went horribly awry. Physicians published case reports detailing the management of septic, radically ill patients who risked their lives procuring illegal abortions (22). As some states liberalized their abortion laws, other researchers focused their work on the public health impacts of safe and legal abortions enabled by better policies, techniques, and antibiotics (23, 24). Their combined efforts eventually pushed professional medical and public health organizations to support abortion rights through advocacy and amicus curiae briefs filed in the United States Supreme Court cases *Roe* and *Casey.*

Legalized abortion opened new research avenues and sparked ethical debates regarding the social and legal complexities of biomedical research during pregnancy. Notably, concerns about the outcome of *Roe* and pressure from anti-abortion groups shaped the first federal “protections” governing research on pregnant patients—regulations first established in the 1970s that excluded pregnant women from clinical trials and created gaps in knowledge about prescription drug use during pregnancy and the postpartum period (25, 26). In recent years, leading research and federal organizations have discussed the need to address these knowledge gaps and have called for a range of studies on reproductive and maternal health needs with an increased emphasis on the social, behavioral, biological, and environmental forces that shape health outcomes at the individual, local, state, and national levels (13, 14). In response to these calls, equity-focused scholars have conducted a range of important studies that prioritize community perspectives and values (27–30).

Research on maternal and reproductive health requires considerable sensitivity, as it often involves meeting people in especially vulnerable moments. For example, studies on stillbirth may require clinicians to approach grieving parents after a pregnancy loss to obtain consent for fetal tissue sampling. Research on maternal morbidity and mortality often necessitates conversations with women after near-death experiences or with families who have lost loved ones in cases of maternal death (31–34). Abortion research similarly involves these weighty social and emotional considerations, in addition to heightened ethical and legal concerns about stigma, confidentiality, trauma, and criminalization. In environments where abortion is criminalized and stigmatized, contemporary research ethics guidelines call for population-sensitive research practices to protect participants and communities that may face threats of persecution or harm (35). Thus, examining how intersectional structures of oppression, stigma, and vulnerability influence abortion research is critical for advancing and informing research ethics practices and protocols in the context of reproductive and maternal health.

### Intersecting structures of oppression and research “vulnerability”

Research ethics guidelines predicated on the assumption of participant autonomy obscure how structural issues threaten reproductive autonomy, perpetuate trauma and stigmatization, and give rise to significant moral distress in groups already burdened by poverty, stigma, and inequitable access to healthcare. Respectful and compassionate research requires an understanding ways in which intersecting, multidimensional structures of oppression shape participant-level vulnerability in research settings. Even in instances where research participants have given informed consent and assumed the individual risks associated with research involving sensitive information, researchers in the post-*Roe* environment have a moral and professional responsibility to grapple with the systems and structures that sharpen participant vulnerability and research risks.

When individuals occupy multiple marginalized identities, they may be rendered more vulnerable in settings where social and structural forces collide to limit their agency, visibility, and voice (36). However, the traditional approach to categorical research protections outlined in the Belmont Report classifies certain groups as vulnerable based on singularly defined identities, namely, incarcerated individuals, children, and people with disabilities. Recent scholarship has expanded the concept of vulnerability to include the intersectional experiences of communities burdened by excessive research risks.

Pregnant women were officially removed as a vulnerable population under the Revised Common Rule in 2017, a shift to ensure that they were justly represented in biomedical research and development and were able to reap the benefits of scientific advancement (37). However, this adjustment preceded the complications posed by the end of the constitutional right to abortion, including threats of bodily harm, stigma, and criminalization. These threats are particularly salient for Black women living in the United States, who are three times more likely to die from preventable pregnancy complications than white women. Racial disparities in maternal health outcomes are amplified by other forms of oppression, such as lack of access to reproductive healthcare, structural racism, and lack of social support, which make women more vulnerable to harm during pregnancy (38). Furthermore, recent estimates indicate that abortion bans have the potential to increase maternal mortality by 21% overall and up to 33% among Black Americans.

Additionally, women who are denied abortions experience a cascade of economic hardships and serious health complications associated with carrying a pregnancy to term (39). Before *Dobbs,* Texas Senate Bill 8 offered a glimpse into the dangerous future of abortion bans and raised questions about which communities were disproportionately harmed by abortion restrictions and increasingly made vulnerable by the research process (6). Previous scholarship reveals that women in minoritized communities may experience excessive research risks and barriers to meaningful research participation because of preexisting comorbidities, environmental factors, and structural inequities (30, 40, 41). These concerns are heightened in states and territories that restrict or ban abortion. Notably, eroding access to abortion care has the most profound and pernicious ramifications for Black families, as Black people are disproportionately burdened by various forms of economic and social inequalities that diminish birth equity and just access to all forms of reproductive healthcare (13, 14).

As an interdisciplinary group of scholars and practitioners with a focus on reproductive health equity, we raise important questions related to power asymmetries between those conducting research and the individuals volunteering as participants. Our concerns include: how might data intended to better understand various birth control methods be safeguarded from surveillance and criminalization? How might vulnerable populations be prioritized in the current political climate? And how might the conceptual frameworks, underlying assumptions, and language used by researchers perpetuate harmful narratives about sexuality, pregnancy, birth control, and abortion?

In light of these questions, we understand research as a powerful tool to advance social justice. We argue that the inclusion of vulnerable groups in research can be a pathway to affirming the rights of all people to partake in social life, public expression, and bodily freedom. Individuals can share invaluable insights derived from navigating their marginalized social positionality, which otherwise may be undervalued, misunderstood, or concealed. Most evidently, research findings can mobilize healthcare systems to better meet the needs of populations who stand to benefit most from new understandings and health innovations. It is in the spirit of balancing these potential benefits and risks that the authors offer these considerations.

### Considerations for ethically responsible abortion research

Abortion restrictions heighten risks for all parties involved in scientific research. However, it is imperative to recognize that research participants are especially vulnerable to research-related harms in the post-*Roe* era. Conducting ethical and respectful abortion research requires investigators to focus on the needs and preferences of marginalized communities across the research continuum, starting with the development of research questions and continuing through the study development, implementation, and dissemination of research findings.

In the absence of formal guidance on abortion-related research ethics, the recommendations that follow have been shaped by the authors’ collective experiences working with structurally vulnerable and disadvantaged populations. The considerations presented in the following sections are intended to highlight the value of meaningful community engagement, dialogue, and collaboration when engaging participants burdened by social and structural vulnerabilities.

#### Community and stakeholder engagement

The equitable and just engagement of individuals and communities in abortion research requires working with community leaders and local organizations to improve ethical decision-making. Sophisticated engagement strategies, especially those that elevate the lived experiences of community members, are critical for understanding and mitigating barriers to reproductive health research participation (9). Community-engaged research prioritizes an iterative, dynamic research process with heightened attention to the needs (i.e., perceived and actual), realities, and experiences of local stakeholders who ultimately shape the research design, implementation, and dissemination of findings (10, 42–44). Notably, community-engaged frameworks shift the emphasis of research away from the benefits received by the research team and instead prioritize the needs and preferences of study participants (45).

Scott, Bray, McLemore, and other scholars highlight the urgent need for collaborative, community-engaged research marked by “radical curiosity and courage” to advance health equity and reproductive justice (27). We follow their lead, embracing cultural humility and meaningful community partnerships, to advocate for a braver, bolder approach to abortion research and reproductive ethics. While traditional research ethics models focus heavily on institutional- and investigator-driven values, we advocate for an expanded understanding of scholarship that accurately reflects and elevates the voices and values of research participants.

#### Risks to participants with social and structural vulnerabilities

Research with communities burdened with social and structural vulnerabilities has given rise to unique ethical challenges that require context-specific research protection and stakeholder engagement. Psychological, legal, social, and economic harms are among the many risks relevant to research in post-Roe environments (28, 46). Volunteers in abortion research may face stigma, criminalization, discrimination, health surveillance, and iatrogenic harms. These considerations are especially applicable to abortion research that employs wastewater metabolite testing, health apps for tracking, and interview and focus group research to understand the experiences of people trying to access abortion (38, 47–49). In light of these risks, researchers should seek guidance from trustworthy stakeholders and local organizations to ensure that their involvement and visibility in the community does not exacerbate risks for already vulnerable groups.

Abortion research participants may be hesitant to disclose the location and state of abortion access because of the potential consequences. Indeed, researchers should evaluate relevant legal risks when working with communities living in areas with restricted abortion access and plan to anonymize or minimize location data collection accordingly. Future research is needed to elicit feedback from community stakeholders to understand how various research settings and social contexts influence the experiences and safety of research participants (11). It is especially important to engage in discourse with community stakeholders to understand their interpretation of the current political landscape as it relates to reproductive healthcare so that researchers can avoid perpetuating harm.

#### Privacy and confidentiality

Prior studies involving individuals with substance use disorders and people who use drugs remind us that privacy and confidentiality concerns are critically important to take into account when data can be used to criminalize and stigmatize individuals and communities (50). Strategies that have been used to enhance privacy and confidentiality include: (1) Certificates of Confidentiality (CoC) which protect the privacy of research participants by restricting access to identifiable, sensitive study information so that it may only be accessed by members of the research team (51); (2) Protocols that require the anonymization and minimization of nonessential sensitive personal health information; (3) Generation of synthetic datasets that mimic the structure and statistical distribution of organically obtained study data while protecting the identity and private health information of the research participants (52); (4) “Shield laws” that protect abortion seekers and their helpers from state interference and other forms of legal harm (53).

Notably, the Department of Health and Human Services (HHS) recently proposed rule changes intended to strengthen the Health Insurance Portability and Accountability Act (HIPAA) Privacy Rule to shield private health information related to pregnancy and reproductive health from law enforcement officials (54). Legislators in some states are discussing broader information privacy laws to protect commercially obtained data such as those collected in period-tracking apps. Some states have passed “shield laws” intended to protect abortion providers, patients, and their helpers, but these laws do not include specific protections for persons involved in abortion research (55). Ultimately, researchers and funding agencies must not only consider how to protect private health information, but also how data generated in abortion research will be communicated and disseminated to the public.

#### Communication and dissemination

Ethical scientific research requires effective communication and timely dissemination of findings to individuals and communities most affected by a particular health issue. Disseminating data to communities is critical for strengthening public trust in clinicians, public health workers, and healthcare systems (56, 57). A thorough, evidence-based understanding of health issues is also integral to advocating for policy changes and interventions that promote reproductive and maternal health equity. This is especially true when a health issue is highly stigmatized or politically charged, as in the case of abortion.

In the current political context, in which abortion research generates partisan divides and purposeful disinformation is rampant, it is critically important to consider how study data are communicated and presented to the public. Ethical attention to abortion research involves engaging trusted community leaders and stakeholders to inform equity-centered research communication. This can be accomplished by developing and committing to communication strategies that outline a plan for if and when research findings are misinterpreted or weaponized against marginalized communities.

## Conclusion

Developing, implementing, and translating ethically sound abortion research policies and procedures calls for concrete and tailored strategies to advance equitable access to scientific discovery and translation. Promoting the ethical inclusion of minoritized groups in reproductive and maternal health research requires specific attention to a myriad of issues, including privacy and fairness in the use of abortion information, informed consent, and the return of results to participants. Further, dedicated attention to the historical realities, contextual challenges, and concerns of diverse research communities is critical to promoting equity in research. Fostering research justice also involves demonstrating optimal respect for reproductive preferences, lived experiences, overlapping social identities, and the moral agency of minority women (15, 58).

Conceptually aligning research with reproductive justice, birth justice, and respectful maternity care frameworks fosters analytic liberation and bolsters scientific rigor (59). Centering equity and respect in research also has salient implications for equipping future scientists, investigators, and clinician scholars with the knowledge, skills, and structural competency to disrupt longstanding oppression in the research enterprise that prevents certain topics from being prioritized, namely those affecting the health and well-being of Black women and other populations made vulnerable by overlapping systems of oppression.

Furthermore, respectful and ethical research highlights the importance of bioethicists with empirical and normative training leading robust discourse around abortion-related research and the healthcare needs of Black women. To safeguard against research-related harms in the post-*Roe* era, it is essential that funding agencies, research institutions, IRBs, and investigators elucidate the needs, values, and preferences of marginalized communities across the research continuum. Insights from existing training programs, funding mechanisms, and organizations are foundational for informing broader research ethics frameworks that responsibly address the complexities that arise in maternal and reproductive health research, especially related to abortion (2, 5, 60). Ethically responsible research in the post-Roe era—especially research with minoritized communities demands equity, justice, and respect.

## Data availability statement

The original contributions presented in the study are included in the article/supplementary material, further inquiries can be directed to the corresponding author/s.

## Author contributions

SS: Conceptualization, Writing – original draft, Writing – review & editing. AA: Writing – review & editing. RD: Writing – review & editing. TM: Writing – review & editing. FL: Writing – review & editing. FF: Conceptualization, Writing – original draft, Writing – review & editing.
